# Long-Term Radiometric Stability of Uncooled and Shutterless Microbolometer-Based Infrared Cameras

**DOI:** 10.3390/s24196387

**Published:** 2024-10-02

**Authors:** Olivier Gazzano, Mathieu Chambon, Yann Ferrec, Guillaume Druart

**Affiliations:** DOTA, ONERA, Université Paris-Saclay, 91120 Palaiseau, France

**Keywords:** long-wave infrared, microbolometer, radiometric calibration, imaging system

## Abstract

Uncooled and shutterless microbolometer cameras are good candidates for infrared imaging systems installed on small satellites or small unmanned aerial vehicles: they are light and passive since no cooling system or mechanical shutter is required and they can be operated at ambient temperatures. However, the radiometric compensation has to be carefully performed to make the system compatible with applications where the radiometric accuracy of the images is mandatory. In this paper, we study the impact of the camera environment to the radiometric accuracy of the images. We propose and test hardware and software solutions to improve this accuracy and the quality of the radiometric images. We show that the radiometric calibration of the camera with our model is valid over a long time period— about 3 years—using in-door experiments.

## 1. Introduction

### 1.1. Context

Long-wave infrared (LWIR) cameras installed on small satellites (nanosatellites) or small unmanned aerial vehicles (sUAVs) can be used for various applications such as Earth surface temperature mapping, wildfire monitoring or urban heat island detection with large satellite revisit frequency. For sUAV systems, smart city applications such as leak detection, buildings thermal efficiency or occupancy and predictive maintenance can also be considered. For such applications, infrared cameras need to fully satisfy the SWaP requirements: small size, small weight and reduced power consumption. For those applications, the image quality and the image temperature calibration stability need to be large enough to meet the application requirements [1].

Semiconductor-based infrared cameras are common LWIR image sensors and they produce high-quality images with high calibration fidelity and temporal stability. They are fully compatible with large satellite systems but they do not meet the SWaP requirements for nanosatellite or sUAV platforms, mostly due to their cryogenic cooling system. Instead, microbolometer-based infrared cameras satisfy the SWaP requirements but the image quality and the image calibration stability can be poor when they are installed on unstable thermal environments such as sUAV or nanosatellite systems [2,3]. A solution is to cool down the microbolometer and the optics; however, this would reduce the SWaP capability of the camera. Another solution is to calibrate the images by using a mechanical shutter. However, this would blind the camera for hundreds of milliseconds, and the shutter is difficult to install on space environments for nanosatellite systems. Moreover, a shutter calibration is a one-point calibration method, meaning that the gain or the offset of the calibration model will have to be temporally stable if good image qualities and reliable image calibrations are required.

In this paper, we will only consider the uncooled—i.e., with no cryogenic nor thermometric cooling (TEC) system attached to the camera—and shutterless microbolometer-based camera for nanosatellite or sUAV payloads [4]. Several papers reported on methods to improve the quality of the images. They are presented in Section 1.2. We will present in Section 2 our hardware and software solutions to reduce the impact of the out-of-scene contribution to the scene signal and describe the experiments we performed to obtain the model parameters. In Section 3, we will introduce the long-term measurements we performed and show the long-term stability of the model. We will then discuss the model and the results in Section 4.

### 1.2. Background

The microbolometer cameras studied in this paper are uncooled, TEC-less and shutterless cameras, making them lighter and more SWaP compatible. However, they are also more sensitive to their thermal environment. The impact of this latter is twofold: first, it modifies the microbolometer chip response, and second, it changes the infrared flux received by the microbolometer from the camera lens and housing. These changes lead to degraded images, with a low image quality and a poor radiometric accuracy.

A standard approach to compensate these image quality losses is to rely on temperature-parameterized tables that can be used for non-uniformity corrections and radiometric calibration procedures. Model parameters can be estimated in the laboratory with a climatic chamber and black-bodies. The full thermal modeling of the systems (electronics, focal plane array, lenses, and housing) is not required in that case. However, temperature sensor locations and correction models have to be determined and used.

It is mandatory to consider the focal plane array (FPA) temperature for TEC-less and shutterless microbolometer-based cameras. The FPA temperature is usually provided by the FPA manufacturer in the header of the image files. Some authors stated that this sole temperature is a good witness of the overall thermal state of the camera and is thus sufficient to parameterize the correction tables. This is, for instance, the assumption made by [5,6,7], and under this hypothesis, P.W. Nugent et al. [6] reached a variability of the estimated scene temperature of 0.2 °C over 24 h. However, to be valid, this assumption requires that the camera is in a very stable state, which limits the operational use of this calibration. J. Kelly et al. [8] tested such cameras in a climatic chamber with simulated wind effects. They pointed out that the calibration is stable between 20 and 60 min. These results were also obtained by Q. Wan et al. [9], who tested their camera in simulated UAV thermal conditions in a very similar way. Furthermore, as stated above, there may be changes between the laboratory conditions and the field environment so that the same FPA temperature is reached for different temperature distributions inside the camera. This can occur if hysteresis effects exist, or is there are other heat sources near the camera (electronics, other instruments, sun radiation, wind, and so on).

For such fully uncooled cameras, the LWIR flux emitted by the inner housing parts will not be negligible compare to the scene signal. A common calculation states that, for F/1 aperture optics, the signal received from the housing is four or five times the signal received from the scene (see Section 2.2, and [10,11]). This means that a 0.25 °C variation in the housing induces the same signal changes as approximately a 1 °C variation in the scene, and as approximately a 20 °C variation in a 5%-absorbent lens if we consider that the scene, the housing and the lens are at approximately the same temperature. Therefore, monitoring and including the housing temperature in the calibration process are required to produce radiometric images.

Such a consideration of the housing temperature was, for instance, proposed by H.M. Qu et al. [12], by A. Wolf et al. [13], who also included the temporal derivative of the temperature, by Y. Cao et al. [14], who complemented the temperature-parameterized non-uniformity correction with image processing, and by A. Tempelhahn et al. [15], who considered housing and ambient temperature effects in the model [16,17,18]. A. Tempelhahn et al. from the Technische Universität Dresden proposed to use three temperature probes inside the camera housing [15,16,19]. This allowed them to manage transition regimes where the camera housing parts have various time constants. They showed that the use of multiple probes gives better results than the use of only one probe with its temporal derivative [15].

The CIRC camera, a compact microbolometer-based LWIR camera developed by Jaxa, was tested on board the Alos-2 satellite. To save mass and power, CIRC was not equipped with a shutter, and ground tests provided a pixel-wise offset, linearly depending on the sensor temperature. In this case, the sensor temperature was the average of the lens and detector package temperatures [20]. However, once in orbit, this calibration strategy gave insufficient results, with a 10 K error on long time period, along with a seasonal variation. This led to the development of a periodic vicarious calibration strategy [21]. The limitations of the ground-based calibration protocol probably come from a difference in the temperature distribution of the lens between orbit and ground-based calibrations [22]. It was initially proposed that this difference was due to the heating of the lens by the laboratory black body. However, the long-term drift and the seasonal variation, as well as the comparison with the second CIRC camera installed in the International Space Station, pointed out more probable external factors such as sun radiation [21]. Therefore, ground conditions need to be thermally identical to the in-flight conditions, and more than one thermal sensor is required for the precise radiometric calibration of the images.

## 2. Materials and Methods

### 2.1. Microbolometer Technology

The pixels of microbolometer cameras are single elements that are sensitive to the received incoming light energy. The incoming light slightly changes the pixel temperature that can be read out by an electronic circuit. To ensure the temperatures changes with the incoming light, each pixel is made of a suspended membrane with a low thermal conductivity with the substrate. The whole sensor is also under vacuum to minimize the heat convection from the environment to the membranes. The membranes are made of amorphous silicon (a-Si) or vanadium oxide (VOx) such that the electrical resistance of the membrane read out by an integrated electronic circuit depends on its temperature [4,19]. The electronic circuit generates the number of digital counts $N_{\mathrm{raw}}$ for each pixel, and this value is linearly proportional to each membrane temperature.

**Figure 1:**
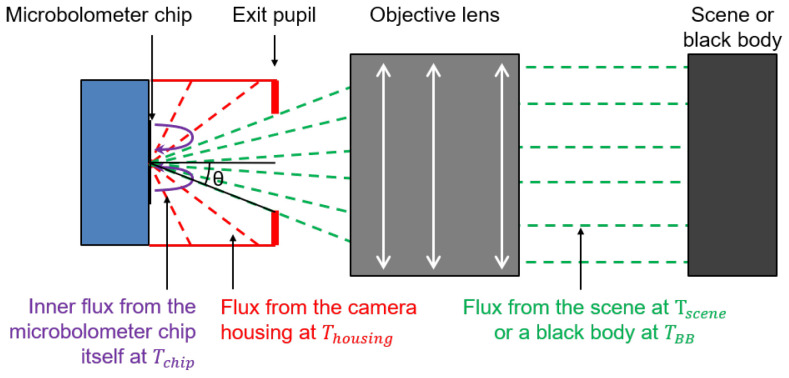
The scene temperature estimated by a microbolometer pixel depends on the scene temperature $T_{\mathrm{scene}}$ but also on the temperature of the microbolometer chip $T_{\mathrm{chip}}$ and on the temperature $T_{\mathrm{housing}}$ of the inner surfaces of the housing between the microbolometer chip and the exit pupil. In the schematic, the optical design is supposed to have a well-defined exit pupil.

In a stable regime, the membrane temperature $T_{i,j}$ for the pixel (i,j) is at a thermal equilibrium between the temperature induced by the optical radiation (of energy $Q_{i,j}$) and a background temperature $T_{i,j}^{0}$. This background temperature is related to the pixel environment such as the FPA temperature due to thermal conduction through the membrane bridges [23] and also to the heat induced by the pixel reading process which applies a bias current across the device’s membrane [24]. We can write:

(1) $$\begin{array}{ccc} T_{i,j} & = & T_{i,j}^{0}+R_{i,j}^{th}\times Q_{i,j} \end{array}$$

where $R_{i,j}$ is the thermal resistance of the membrane of the pixel (i,j). The term $Q_{i,j}$ depends on the scene temperature $T_{\mathrm{scene}}$ but also on a background signal that depends on the temperature $T_{housing}$ of the housing parts that are in front of the camera (Section 2.2, Figure 1 and [19]).

### 2.2. Impact of the Radiation of the Camera Housing on the Measured Signal

The thermal resistance of a microbolometer pixel and the number of digital counts $N_{\mathrm{raw}}$ measured by the camera depend on the radiance ${\hat{L}}_{\mathrm{chip}}$ due to the chip at the temperature $T_{\mathrm{chip}}$, on the radiance of the signal of the scene ${\hat{L}}_{\mathrm{scene}}$ and of the housing parts in the surrounding between the microbolometer chip and the exit pupil ${\hat{L}}_{\mathrm{housing}}$ (Figure 1). We can write that:

(2) $$\begin{array}{ccc} N_{\mathrm{raw}} & = & N_{0}+g\times \left({\hat{L}}_{\mathrm{scene}}+{\hat{L}}_{\mathrm{chip}}+{\hat{L}}_{\mathrm{housing}}\right) \end{array}$$

where *g* is a gain term and $N_{0}$ is a background signal that depends on the chip temperature. For a scene with an emissivity of ϵ(λ) at a wavelength λ and a surface temperature $T_{\mathrm{scene}}$, for a microbolometer camera with a spectral response η(λ) and for a surrounding housing with a spectral emissivity of $\epsilon_{\mathrm{housing}}\left(\lambda \right)$ and a surface temperature $T_{\mathrm{housing}}$, we have this relation between temperature *T* and spectral radiance *L*:

(3) $$\begin{array}{ccc} L_{\mathrm{scene}}\left(T_{\mathrm{scene}}\right) & = & \int_{0}^{\infty }\epsilon_{\mathrm{scene}}\left(\lambda \right)\cdot P\left(\lambda ,T_{\mathrm{scene}}\right)\cdot \eta \left(\lambda \right)d\lambda \\ L_{\mathrm{housing}}\left(T_{\mathrm{housing}}\right) & = & \int_{0}^{\infty }\epsilon_{\mathrm{housing}}\left(\lambda \right)\cdot P\left(\lambda ,T_{\mathrm{housing}}\right)\cdot \eta \left(\lambda \right)d\lambda \\ L_{\mathrm{chip}}\left(T_{\mathrm{chip}}\right) & = & \int_{0}^{\infty }\epsilon_{\mathrm{chip}}\left(\lambda \right)\cdot P\left(\lambda ,T_{\mathrm{chip}}\right)\cdot \eta \left(\lambda \right)d\lambda \end{array}$$

where P is the Planck’s law in energy (radiance):

(4) $$\begin{array}{c} P\left(\lambda ,T\right)=\frac{2hc^{2}}{\lambda^{5}}\frac{1}{\exp\left(\frac{hc}{\lambda k_{B}T}\right)-1}, \end{array}$$

where *h* is the Planck constant, *c* the speed of light in the medium and $k_{B}$ the Boltzmann constant. We can see from Equations (2) and (3) that the housing part temperature will have an impact on the measured signal. To more quantitatively evaluate the impact of this effect on the image radiometric quality, we established a simple radiometric model of the camera to compare the flux to a central FPA pixel from the scene ${\hat{L}}_{\mathrm{scene}}$ and the flux from the inner housing parts ${\hat{L}}_{\mathrm{housing}}$ [25]. In the case of an optical design with an exit pupil, we can show that the received fluxes on the central pixel, for a uniform scene at a temperature $T_{\mathrm{scene}}$ and emissivity of 1, are proportional to:

(5) $$\begin{array}{ccc} {\hat{L}}_{\mathrm{scene}}\left(T_{\mathrm{scene}}\right) & \propto & s^{2}\cdot \pi \cdot t_{\mathrm{opt}}\cdot L_{\mathrm{scene}}\left(T_{\mathrm{scene}}\right)\cdot \sin^{2}\left(\theta_{\mathrm{pupil}}\right)\\ {\hat{L}}_{\mathrm{housing}}\left(T_{\mathrm{housing}}\right) & \propto & s^{2}\cdot \pi \cdot \left(\alpha_{4}\cdot L_{\mathrm{housing}}\left(T_{\mathrm{housing}}\right)+\alpha_{5}\cdot L_{\mathrm{housing}}^{2}\left(T_{\mathrm{housing}}\right)\right)\cdot \left(1-\sin^{2}\left(\theta_{\mathrm{pupil}}\right)\right)\\ {\hat{L}}_{\mathrm{external}}\left(T_{\mathrm{opt}},T_{\mathrm{housing}}\right) & = & {\hat{L}}_{\mathrm{scene}}\left(T_{\mathrm{opt}}\right)+{\hat{L}}_{\mathrm{housing}}\left(T_{\mathrm{housing}}\right) \end{array}$$

where $\theta_{\mathrm{pupil}}=\arctan\left(1/\left(2N\right)\right)$ and *N* is the f-number of the lens. The term *s* is the pixel size and $t_{\mathrm{opt}}$ is the transmission of the optical components. $\alpha_{4}$ and $\alpha_{5}$ are coefficients related to the coefficients $a_{4}$ and $a_{5}$ that will be introduced in Equation (6).

In order to find the impact of a 1 °C increase in the housing’s temperature at room temperature, we need to calibrate the modeled system. To do so, we consider that the scene is a perfect black body with $\epsilon_{\mathrm{scene}}=1$ at the temperature $T_{\mathrm{scene}}$. We also calculate ${\hat{L}}_{\mathrm{external}}$ for $T_{\mathrm{housing}}=20$ °C, $\epsilon_{\mathrm{housing}}=0.76$ and for a set of $T_{scene}$ within the range [−10,60] °C. By numerical interpolations, this allows us to calibrate the model and establish a link between ${\hat{L}}_{\mathrm{scene}}\left({\tilde{T}}_{\mathrm{scene}}\right)$ and the estimated temperature of the scene ${\tilde{T}}_{\mathrm{scene}}$. Using this calibration and Equation (5), we find that an increase of 1 °C in $T_{\mathrm{housing}}$ leads to an increase of 4.6 °C in the estimated scene temperature ${\tilde{T}}_{scene}$. This value is validated experimentally as discussed in Section 4.

The impact of a change in the temperature of the inner parts of the housing is then significant on the measured signal and it needs to be accounted for to ensure the reliable radiometric qualities of the images. It also needs to be considered for the geometric quality of the images because of the non-uniform radiance of the inner housing parts.

Using an empirical approach with the experiments described later in this section, we find that ${\hat{L}}_{\mathrm{housing}}\left(x,y\right)$ for the pixel of coordinates (x,y) equals:

(6) $$\begin{array}{ccc} {\hat{L}}_{\mathrm{housing}}\left(x,y\right) & = & a_{4}\left(x,y\right)\cdot L_{\mathrm{housing}}+a_{5}\left(x,y\right)\cdot L_{\mathrm{housing}}^{2} \end{array}$$

where $a_{4}$ and $a_{5}$ are matrices of constant coefficients for every pixel (x,y) of the sensor. As shown experimentally in Section 3.2, this model—that considers ${\hat{L}}_{\mathrm{housing}}$ as a second order polynomial function of the integrated spectral radiance $L_{\mathrm{housing}}$ (Equation (6))—leads to good data fitting.

### 2.3. Experimental Setup

**Figure 2:**
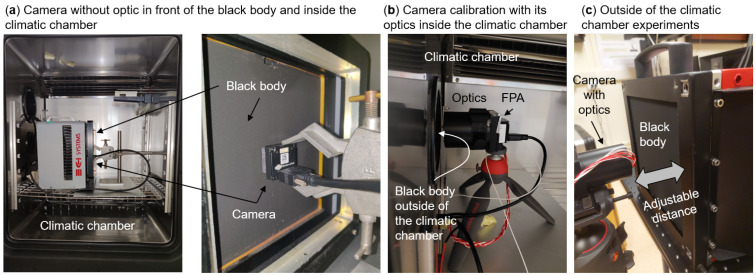
Experimental setups used in this paper as explained in Section 2.3 of the main text.

Depending on the experiments we perform, we can install the camera inside the climatic chamber without its optics and any mechanical front housing. In that case, the camera is installed a few millimeters in front of the black body (Figure 2a). This allows us to adjust the black body temperature and the chip temperature independently and retrieve the offset and the gain of the FPA (Section 3.1).

We can also install the camera with its optics inside the climatic chamber in front of a black body (Figure 2b). In that case, it is more convenient to install the black body outside the climatic chamber. We isolated the black body surface with the climatic chamber so as not to degrade the climatic chamber performances. We used this setup to calibrate the camera by building acquisition look-up tables (Section 3.2).

To measure the effect of the black body–camera distance (Section 3.3) and to prove the long-term stability of the model (Section 3.4), we can install the camera with its optics and the black body outside the climatic chamber on a laboratory bench (Figure 2c). We can adjust the distance between the camera and the black body surface from a few millimeters up to about 2.5 m. The focus is set at about 1.5 m.

## 3. Results

### 3.1. Chip Temperature-Dependent Gain

**Figure 3:**
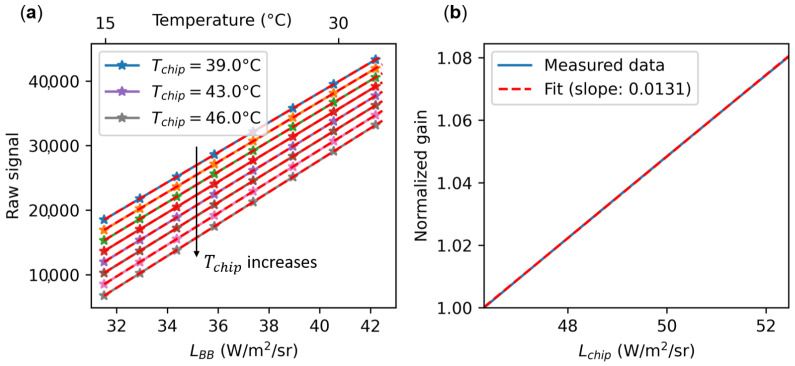
The gain depends on the temperature of the microbolometer matrix. (**a**) Raw signal of the central pixel of the microbolometer camera as a function of the black body radiances $L_{\mathrm{BB}}$ and for several microbolometer chip temperatures $T_{\mathrm{chip}}$. The raw signals were interpolated from experimental data to have $T_{\mathrm{chip}}$ on a regularly spaced grid. The gain is defined as the slope of the raw signal as a function of $L_{\mathrm{BB}}$. Note that the upper scale in temperature is not linear with $L_{\mathrm{BB}}$. (**b**) Obtained gain as a function of $L_{\mathrm{chip}}$. It increases approximately by 1 percentage point per degree Celsius.

The gain term *g* in Equation (2) converts the received radiances to the digital raw counts of the corresponding pixels. This subsection is dedicated to estimating the impact of the chip inner radiance $L_{\mathrm{chip}}$ to the gain term. We found using an empirical approach based on data fitting (Figure 3b) that the gain term depends linearly on the chip spectral radiance rather than on the chip temperature:

(7) $$\begin{array}{ccc} g(x,y) & = & g_{0}\left(x,y\right)+g_{1}\left(x,y\right)\times L_{\mathrm{chip}} \end{array}$$

In order to prove this linear dependence, we performed experiments on a microbolometer camera without the optics and without any camera housing parts in front of the FPA. We installed the camera in front of a black body inside a climatic chamber (Figure 2a). This allowed us to adjust the camera chip temperature $T_{\mathrm{chip}}$ and to calculate $L_{\mathrm{chip}}$ according to Equation (3) considering that $\epsilon_{\mathrm{chip}}=1$. By changing the black body temperature $T_{\mathrm{BB}}$ for several climatic chamber temperatures, we measured the raw signal on the central pixel of the camera as a function of $T_{\mathrm{chip}}$ and $T_{\mathrm{BB}}$. By interpolating the raw signal as a function of $T_{\mathrm{BB}}$ for some $T_{chip}$ values, we obtained Figure 3a.

For a given $T_{\mathrm{chip}}$ (related to $L_{\mathrm{chip}}$ considering Equation (3)), the gain *g* is the slope of the raw signal as a function of the radiance $L_{\mathrm{BB}}$ of a black body at a temperature $T_{\mathrm{BB}}$. Using a linear fitting, we obtain the gain $g(L_{\mathrm{chip}})$ as a function of the spectral radiance of the chip $L_{\mathrm{chip}}$. The normalized gain $g\left(L_{\mathrm{chip}}\right)/g\left(L_{\mathrm{chip}}^{0}\right)$ (with $L_{\mathrm{chip}}^{0}=46.3\;$W/m^2^/sr; i.e., the lowest $L_{\mathrm{chip}}$ measured) is plotted in Figure 3b. The normalized gain as a function of $L_{\mathrm{chip}}$ significantly increases by 8% for an increase of 6 W/m^2^/sr of $L_{\mathrm{chip}}$ or approximately 1 percentage point per degree Celsius (using Equation (3)). The dependence is fairly linear as a function of $L_{\mathrm{chip}}$, and thus it validates Equation (7) where $g_{1}$ is a coefficient that depends on the pixel number and that equals $0.0131\times g_{0}$ (slope of the curve in Figure 3b) in this particular case for the central pixel of the microbolometer camera used (see Figure 3).

This temperature dependence of the gain has also been reported with similar values [16,26] but some other authors did not observe it [14,17,27]. Such different observations may be explained by differences in the microbolometer material, in the readout circuit design or in the operating camera parameters.

### 3.2. Correction Model and Radiometric Image Calibration

In this subsection, we propose two models—a traditional one that does not consider the radiance of the camera housing and one model that does—in order to estimate the raw counts on the camera.

According to Section 2.2 and Section 3.1, we can write the raw counts on the optical sensor as:

(8) $$\begin{array}{c} N_{\mathrm{raw}}^{\mathrm{model}\;1}\left(x,y\right)=a_{0}+\left(a_{1}+a_{2}\cdot L_{\mathrm{chip}}\right)\times \left(L_{\mathrm{scene}}+a_{3}\cdot L_{\mathrm{chip}}\right)\\ N_{\mathrm{raw}}^{\mathrm{model}\;2}\left(x,y\right)=a_{0}+\left(a_{1}+a_{2}\cdot L_{\mathrm{chip}}\right)\times \left(L_{\mathrm{scene}}+a_{3}\cdot L_{\mathrm{chip}}+a_{4}\cdot L_{\mathrm{housing}}+a_{5}\cdot L_{\mathrm{housing}}^{2}\right) \end{array}$$

where all $a_{i}$ coefficients depend on the pixel number (x,y) and are different for the two models. Equation (8) refers to model 1 that does not account for the radiance of the housing while Section 3.2 refers to model 2 that does (Section 2.2).

In order to find the four (model 1) and six (model 2) $a_{i}$ coefficients for every pixel of the microbolometer FPA, we installed the full camera (with its optical lens) inside the climatic chamber in front of a black body of surface temperature $T_{\mathrm{BB}}$ and emissivity near unity within the microbolometer spectral domain (Figure 2b). For the purpose of model 2, we mounted four temperature sensors (PT100) inside the optical mounts of the camera optics to monitor $T_{\mathrm{housing}}$ (mean value of the four temperature sensors). We also installed a flexible resistive heater around the optical mount in order and a thermal insulator between the FPA case and the optics housing to slightly decouple $T_{\mathrm{housing}}$ from $T_{\mathrm{chip}}$.

**Figure 4:**
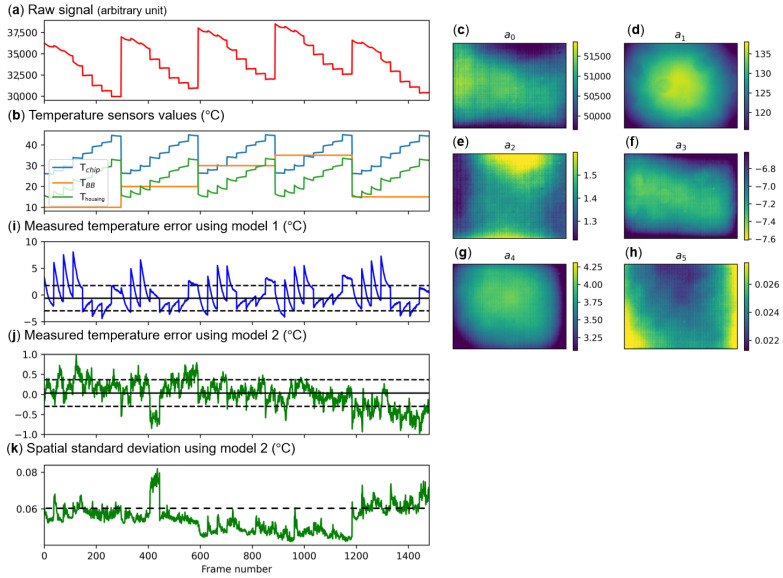
Radiometric calibration. (**a**) Raw signal on the central pixel of the microbolometer camera as a function of the frame number. (**b**) Temperatures of the microbolometer chip $T_{\mathrm{chip}}$, of the black body installed in front of the camera $T_{\mathrm{BB}}$ and of the housing mounts of the optics $T_{\mathrm{housing}}$. (**c**–**h**) Six coefficient maps $a_{i}\left(x,y\right)$ of Section 3.2 for every pixel (x,y) of the camera obtained from (**a**,**b**) by least-square fittings. (**i**,**j**) Difference between the estimated temperature obtained from the calibrated data of the central pixel and $T_{\mathrm{BB}}$ for model 1 (without considering $T_{\mathrm{housing}}$, (**i**)) and model 2 (considering $T_{\mathrm{housing}}$, (**j**)). (**k**) Spatial standard deviation using model 2 over the full matrix for every frame.

Figure 4a shows the raw signal on the central pixel of the microbolometer as a function of the frame number. The frame number corresponds to various temperatures of the black body $T_{\mathrm{BB}}$, of the microbolometer chip $T_{\mathrm{chip}}$ and of the camera housing mounts $T_{\mathrm{housing}}$ (Figure 4b) while changing the black body and climatic chamber set temperatures and by applying for some frames a current through the flexible resistive heater. We note that only frames right after the resistive heater is turned off are displayed in the figure and used for the fitting in order to only operate on quasi-equilibrium images.

The four coefficients for model 1 and the six coefficients for model 2 are obtained per pixel of the matrix by fitting, with a least-squares method, the raw signal to the corresponding black body radiance $L_{\mathrm{BB}}\left(T_{\mathrm{BB}}\right)=\int_{0}^{\infty }P\left(\lambda ,T_{\mathrm{BB}}\right)\cdot \eta \left(\lambda \right)d\lambda$. The scene is supposed to be uniform and such that $L_{\mathrm{scene}}\left(x,y\right)=L_{\mathrm{BB}}\left(T_{\mathrm{BB}}\right)$. The six coefficients we obtained for model 2 are displayed in Figure 4c–h.

To check the completeness and quality of the model, we applied the inverse model to the data themselves using Section 3.2 describing model 2 to find the measured radiance of the scene with the two models:

(9) $$\begin{array}{ccc} {\tilde{L}}_{\mathrm{scene}}^{\mathrm{Model}\;1} & = & \frac{N_{\mathrm{raw}}^{\mathrm{model}\;1}\left(x,y\right)-a_{0}}{a_{1}+a_{2}\cdot L_{\mathrm{chip}}}-a_{3}\cdot L_{\mathrm{chip}}\\ {\tilde{L}}_{\mathrm{scene}}^{\mathrm{Model}\;2} & = & \frac{N_{\mathrm{raw}}^{\mathrm{model}\;2}\left(x,y\right)-a_{0}}{a_{1}+a_{2}\cdot L_{\mathrm{chip}}}-a_{3}\cdot L_{\mathrm{chip}}-a_{4}\cdot L_{\mathrm{housing}}-a_{5}\cdot L_{\mathrm{housing}}^{2} \end{array}$$

and we converted the results from radiance to temperature using Planck’s law (Figure 4i,j). For model 1 (Figure 4i), the difference of the measured temperature to the black body temperature was large, with a median error of −0.60 °C and a standard deviation error of 2.29 °C. When the six coefficients were used (model 2, Figure 4j), we found a median error of 0.03 °C and a standard deviation of 0.32 °C. With model 2, we also found that the spatial standard deviation was 0.06 K, showing the good spatial quality of the images after the correction (Figure 4k).

The better results with model 2 and the six parameters emphasize the need to consider the temperature of the housing on the model. The model 2 results also show the good quality of the model on the calibration data themselves.

### 3.3. Reduction of the External Environment Background

This section shows the need to account for out-of-field-of-view scene signals on the calibration model and in particular for experiments where the black body is far from the camera (the opposite configuration of the inside the climatic chamber scenario).

**Figure 5:**
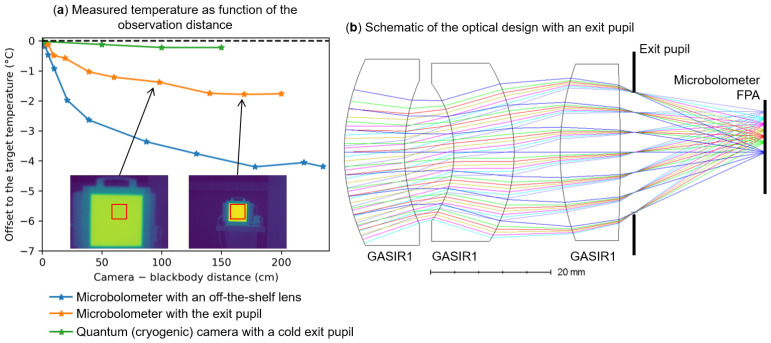
Effect of stray light to the microbolometer signal. (**a**) Offset of the black body measured temperature to the set value of the back body temperature as a function of the distance between the black body and the camera, $d_{bb-c}$, for two kinds of cameras (microbolometer-based and quantum-based) and two kinds of optical lens designs (with an off-the-shelf lens or with a lens with exit pupil). We notice an increase in the signal at small distances for all cameras but mostly for the microbolometer camera with an off-the-shelf lens (see main text). The black body has a temperature that is 15 °C above room temperature (25 °C). (**b**) Our design of the lens with an exit pupil. The objective lens was fabricated by Umicore using a GASIR1^®^ chalcogenide glass.

Figure 5a plots the measured temperature as a function of the distance $d_{bb-c}$ between a black body and three different LWIR cameras (two microbolometer-based cameras with different kinds of lens designs and a quantum-based infrared camera; Figure 2c). For clarity, the temperature of the back body is subtracted to the displayed values. The black body has a temperature that is 15 °C above room temperature (25 °C). With a commercial standard lens with only an entrance pupil (blue curve), we notice that the measured temperature decreases by almost 4 °C when $d_{bb-c}$ goes from a few millimeters to almost 2 m. This offset bias is far above the requirements for many applications that requires radiometric images.

This decrease is due to unwanted background flux and out-of-field-of-view signals that are reflected or scattered inside the objective lens and reach out to the microbolometer FPA. It can also be due to light from the field of view that is scattered by dust on the lens surfaces. For large $d_{bb-c}$, most of the unwanted signal arises from the room temperature (at approximately 25 °C). When the black body is close, most of the unwanted signal comes from the black body emission (40 °C). Due to the larger black body temperature than the room temperature, the estimated temperature by the microbolometer decreases with longer distances.

To reduce the impact of unwanted light, we designed a new lens with an exit pupil (Figure 5b). Such design blocks more stray light than the previous optical design. Indeed, we notice a strong improvement on the offset at low distances (orange curve in Figure 5a). The position of the pupil, the lens surface quality and the optical baffle efficiency are then crucial aspects for the radiometric quality of microbolometer-based infrared cameras.

To compare with a camera with a cold exit pupil and with a lens with a lower aperture, we performed the experiments with a FLIR quantum-based LWIR camera (aperture F/2.0). For this camera, the exit pupil is cooled down to a cryogenic temperature using the cold finger of the FPA. We notice in Figure 5a a small distance dependence. This is because of the small aperture of the cameras and because the pupil is cooled down to minimize its infrared radiation.

### 3.4. Validation of the Long-Term Calibration Model outside the Climatic Chamber

In order to prove the quality of model 2 on another set of data outside the climatic chamber, we performed more than 900 min of image acquisitions over 6 different days from July 2021 to May 2024 (Table 1). We installed the camera in a laboratory bench, outside the climatic chamber, in front of a black body (Figure 2c). The camera was installed about 1.5 m away from the black body such that the black body active surface corresponds to about one quarter of the field of view of the camera. This distance is significantly longer than in the climatic chamber where they are only a few centimeters apart to cover more than the camera field of view.

According to Section 3.3 and Figure 5a, we need to account for the background signal on the calibration model since the black body is very close to the camera in the climatic chamber and not for the bench experiment. One can easily see that the gain term *g* and the background signal $N_{0}$ in Equation (2) outside and inside the climatic chamber are related by:

(10) $$\begin{array}{ccc} g^{ouside} & = & b\times g^{inside}\\ N_{0}^{ouside} & = & c+N_{0}^{inside} \end{array}$$

where *b* and *c* are constant terms that apply for the whole FPA array. Therefore, this means that experimentally we need to find those two extra parameters, *b* and *c*, in order to obtain a radiometric calibration of the images from the climatic chamber calibration data sets. Those two parameters can be obtained by imaging two small black bodies to cover only a few pixels of the field of view at two different temperatures. In our case, we performed this calibration for the measurement sets number ① and ② in Table 1, and we used those values for the other measurements. By inverting Section 3.2 and using Equation (10), we obtain for model 2:

(11) $$\begin{array}{ccc} L_{\mathrm{scene}} & = & \frac{1}{b}\cdot \frac{N_{\mathrm{raw}}-a_{0}-c}{a_{1}+a_{2}\cdot L_{\mathrm{chip}}}-a_{3}\cdot L_{\mathrm{chip}}-a_{4}\cdot L_{\mathrm{housing}}-a_{5}\cdot L_{\mathrm{housing}}^{2} \end{array}$$

**Figure 6:**
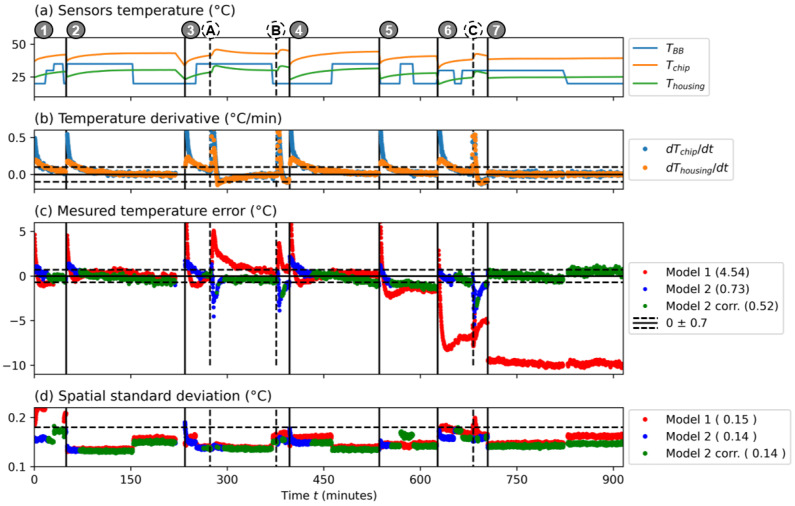
Validation of the radiometric calibration model outside of the climatic chamber. (**a**) Black body temperature $T_{\mathrm{BB}}$, chip temperature $T_{\mathrm{chip}}$ and housing temperature $T_{\mathrm{housing}}$ as a function of time. Measurement numbers ① to ⑦ and heating events Ⓐ to Ⓒ are described in Table 1. (**b**) Temporal derivative of $T_{\mathrm{BB}}$ and $T_{\mathrm{chip}}$ as a function of time. The horizontal dotted line represents 0.1 °C/min. (**c**) Measured temperature error to the black body temperature. Model 1 (red) and model 2 (blue) are considered. Post-selected data (Model 2 corr) where $dT_{\mathrm{chip}}/dt<0.1$ °C/min and $T_{\mathrm{housing}}<0.1$ °C/min are plotted in green. Numbers in the figure caption are temporal standard deviation values over the data set. (**d**) Spatial standard deviation for the data sets of (**c**). Numbers in the figure caption are median values over the corresponding data set.

Experimentally, we changed from time to time the black body temperature for measurements ① to ⑦ in Table 1 (Figure 6a). Moreover, for measurements ③ and ⑥, we performed external heating events with a heat gun.

We can apply the corrected calibration model to the whole 900 min of experiments and compare the estimated temperature of a pixel to the back body actual temperature (Figure 6c) considering model 1 (red) and model 2 (blue). We can see the significant differences between the two models. The standard deviation error over all the data points is 4.54 °C for model 1 and 0.73 °C for model 2, showing the advantage of considering the housing temperature within the model for the quality of the radiometric calibration. The results are also closer to the expected temperatures for all the measurements, especially for measurements ⑤, ⑥ and ⑦ that we performed several months after the calibration processes. The results are also less dependent on the external heating events (quick warming up of the lens with a heat gun, events Ⓐ, Ⓑ and Ⓒ; Figure 6a). Finally, we notice that the spatial standard deviation within the black body region is almost identical in the two models, ranging from 0.15 °C to 0.14 °C (Figure 6d).

We still notice significant deviations from the expected temperatures when the camera temperatures are not stable enough, such as after camera warming-up (i.e., after switching it on) and after the heating events. Therefore, in order to consider only quasi-equilibrium data, we post selected frames where the absolute value of the temperature derivative of the chip temperatures ($T_{\mathrm{chip}}$) and of the housing temperature ($T_{\mathrm{housing}}$) are smaller than 0.1 °C/min (see Figure 6b). In that case, the standard deviation on the measured temperature error is reduced down to 0.52 °C (Figure 6c, green curve), meaning that the radiometric quality of the images becomes more reliable under stable environments and that the monitoring of camera temperature sensors can be useful to check the accuracy of the estimated temperatures.

## 4. Discussion

We have shown that the standard deviation of the measured temperatures is 0.52 °C when the camera temperature is stable enough ($dT_{\mathrm{chip}}/dt$ and $dT_{\mathrm{housing}}/dt<0.1$ °C/min). This value makes the camera compatible with infrared thermographs for fever screening where an accuracy lower than the 0.5 °C is required [28]. In the international standard of [29], the environment needs to be stable (between 18 °C and 24 °C, clause 201.5.3; and airflow from ventilation ducts should be deflected to minimize forced cooling or heating of the target, clause 201.7.9.3.9): the <0.1 °C/min thermal stability required here should be satisfied.

**Figure 7:**
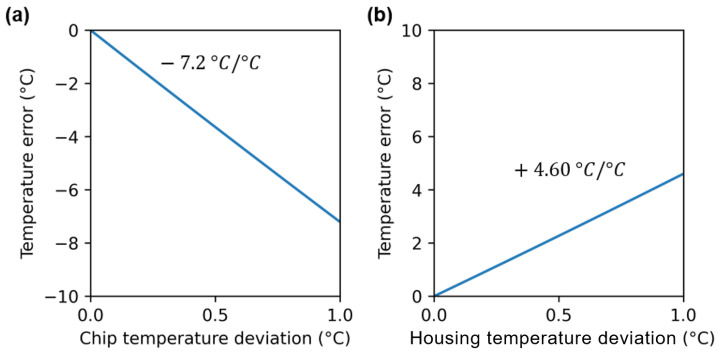
Dependence of the measured temperature as a function of the chip temperature (**a**) and of the temperature of the housing parts between the chip and the pupil (**b**).

On another aspect, from the $a_{i}$ coefficients of Figure 4 and Equation (11), we can calculate the temperature dependence of the error on the estimated scene temperature (equivalent black body temperature) as a function of the temperature of the chip $T_{\mathrm{chip}}$ (Figure 7a) and of the housing $T_{\mathrm{housing}}$ (Figure 7b). As seen in Section 3, Figure 7 illustrates the need for the precise monitoring of the chip and the housing part temperatures. Indeed, Figure 7 shows that a 1 °C increase in $T_{\mathrm{chip}}$ leads to a decrease by 7.2 °C in the measured temperature and that a 1 °C increase in $T_{\mathrm{housing}}$ leads to an increase by 4.6 °C in the measured temperature. This is in agreement with our theoretical predictions when considering $\epsilon_{\mathrm{housing}}=0.76$ (Section 2.2).

In addition, we have shown in Section 3.3 the impact of the environment background on the camera signal, which reduces the estimated scene temperature reliability. This is mostly due to the stray light flux that can arise from multiple effects such as scattering or reflections on the lens mount wall, the scattering of the lens surfaces or a back reflection from the FPA and the last lens’s almost flat surface (Figure 5). We proposed and tested an optical and housing design in Section 3.3 to reduce this effect by a factor of almost two. It could be further increased by choosing a surface material (painting or coating, for instance) with an emissivity closer to unity in the inner housing parts between the chip and the pupil. This would reduce the stray light of the mounts reflected by those materials and improve the reliability of the correction model introduced in Section 3.2 and Section 3.4, and then of the estimated scene temperatures.

## 5. Conclusions

In conclusion, we studied the impact of various parameters on the images radiometric quality taken by a shutterless and an uncooled microbolometer camera. We proposed a solution to consider the temperature of the housing of the lens in addition to the traditional parameters such as the chip temperature. We validated this solution indoors over a long time period. Then, we showed that the use of the housing temperature on the model makes the radiometric correction less sensitive to the camera environment. We also proposed temporal stability criteria on the housing and FPA temperatures in order to improve the fidelity of the calibration model. This may apply to outdoor experiments. We also showed that the gain of the radiometric calibration model is sensitive to the microbolometer chip temperature.

We also proposed a solution to partially reduce the impact of the stray light signal using an optical design with an exit pupil. This reduction could be further improved in the future and we showed that it is reliable for precise radiometric measurements as hot objects in or outside the field of view could reduce the estimated temperature reliability. This will be further required for microbolometer-based cameras installed on an sUAV or a nanosatellite payload because the stray light flux will be mostly unknown and fluctuating.

## Figures and Tables

**Table 1:** Experimental validation of the long-term stability of the calibration model. Measurements date and duration are indicated. The names of the heating events of the microbolometer optics using an air-based heat gun are written in the corresponding last column. These experimental data are used to check the radiometric correction model to significant temperatures changes of the camera optical housing and FPA chip. The events Ⓐ to Ⓒ indicate the measurements for which we heated the camera with a heat gun to change the camera temperature and test the model on harder temperature conditions.

Measurement Number	Measurement Date	Measurement Duration	Heating Events
①	7 July 2021	50 min	-
②	15 July 2021	184 min	-
③	16 July 2021	160 min	Ⓐ and Ⓑ
④	19 July 2021	140 min	-
⑤	26 November 2021	90 min	-
⑥	17 April 2023	76 min	Ⓒ
⑦	17 May 2024	211 min	-

## Data Availability

The original contributions presented in the study are included in the article, further inquiries can be directed to the corresponding author.
